# Supplementary material for: Seroprevalence and risk factors of hantavirus and hepatitis E virus exposure among wildlife farmers in Vietnam
Source: PLoS One. 2025 Aug 7;20(8):e0329570. doi: 10.1371/journal.pone.0329570 (PMC12331095; doi:10.1371/journal.pone.0329570)
Supplement: S1 File — (PDF) [file pone.0329570.s001.pdf]

WILDLIFE VALUE CHAIN AND TRANSMISSION RISK FACTORS SURVEY

A	PRE-INTERVIEW SECTION			
NO.	QUESTION	RESPONSE	OPT	SKIP
A1	Consent form was administered and signed	No<br>Yes	0<br>1	0 → END
A2	Date of interview	*(Automatically generated)*		
A3	Respondent's ID		[ ]	
A4	Name of interviewee		[ ]	
A5	Name of interviewer		[ ]	
A6	Begin time of the interview	*(Automatically generated)*		
A7	Province	Lao Cai<br>Dong Nai	1<br>2	
A8	District	Lao Cai City<br>Bao Yen<br>Bao Thang<br>Bien Hoa City<br>Dinh Quan<br>Vinh Cuu<br>Tan Phu<br>Other<br>*(specify):* _______	1<br>2<br>3<br>4<br>5<br>6<br>7<br>-77<br>[ ]	
A9	Commune	*(specify):* _______	[ ]	

B	DEMOGRAPHICS SECTION OF WILDLIFE FARMERS AND CONSUMERS			
NO.	QUESTION	RESPONSE	OPT	SKIP
B1	What is the respondent's gender?<br>*Interviewer: Do not ask – record as the respondent's appearance*	Male<br>Female	1<br>2	
B2	What is your marital status?	Married<br>Single<br>Divorced<br>Widowed<br>Other<br>*(specify):*________	1<br>2<br>3<br>4<br>-77<br>[ ]	
B3	What is your ethnicity?	Kinh<br>Dao<br>H'Mong<br>Tay<br>Thai<br>Muong<br>Other<br>*(specify):* ________	1<br>2<br>3<br>4<br>5<br>6<br>-77<br>[ ]	
B4	In what year you were born? (yyyy for example 1982)	*(Type in: Numeric only)*<br>yyyy	[ ]<br>-99	

B5	What is the highest level of education that you have completed?<br><br>*Interviewer:*<br>*The highest level of education completed according to the formal twelve-year education system, write 0 if they did not go to school, write 13 if they completed higher educational level after grade twelve (college, university,…)*	Highest level of education completed<br>Do not know	[ ]<br>-99	
B6	What is your position on the farm?	Owner<br>Hired laborer<br>Other<br>*(specify):* ________	1<br>2<br>-77<br>[ ]	
B7	What is the distance between your house and the farm?	_______ (m)	[ ]	
B8	What wildlife species are you currently raising?<br><br>*Interviewer:*<br>• *Select ALL that apply*	Bats<br>Bamboo rats<br>Asian palm civets<br>Masked palm civets<br>Wild Boars/Native Pigs<br>Snakes<br>Porcupines<br>Pythons<br>Crocodiles<br>Other<br>*(specify)* ______	1<br>2<br>3<br>4<br>5<br>6<br>7<br>8<br>9<br>-77<br>[ ]	
B9	Besides the above wildlife species, do you raise any other animals?<br><br>*Interviewer:*<br>• *Select ALL that apply*	Do not raise any other animal<br>Buffaloes<br>Cows<br>Pigs<br>Chickens<br>Ducks<br>Muscovy ducks<br>Goats<br>Sheep<br>Dogs<br>Cats<br>Other<br>*(specify)* ______	0<br>1<br>2<br>3<br>4<br>5<br>6<br>7<br>8<br>9<br>10<br>-77<br>[ ]	
B10	Can domestic animals (buffalo, cow, pig, chicken, duck, goose, goat, sheep, etc.) come into direct contact with wild animals?	No<br>Yes<br>Do not know	0<br>1<br>-99	
B11	Are pets allowed in the wildlife enclosure area (including food and bedding storage areas)?	No<br>Yes<br>Do not know	0<br>1<br>-99	
B12	When did you start wildlife farming activity?	yyyy<br>Do not remember/know	[ ]<br>-99	
B13	Besides wildlife farming, do you have any other occupations?	No other occupation<br>State employee<br>Private company	0<br>1<br>2	

	Interviewer:<br>• *Select ALL that apply*	Trading/self-employed<br>Farming/crop cultivation<br>Livestock and poultry farming<br>Other<br>*(specify)* ______	3<br>4<br>5<br>-77<br>[ ]	
B14	Which activity takes up the most time?	State employee<br>Private company<br>Trading/self-employed<br>Farming/crop cultivation<br>Livestock and poultry farming<br>Wildlife farming<br>Other<br>*(specify)* ______	1<br>2<br>3<br>4<br>5<br>6<br>-77<br>[ ]	
B15	Which job brings in the highest income for you	State employee<br>Private company<br>Trading/self-employed<br>Farming/crop cultivation<br>Livestock and poultry farming<br>Wildlife farming<br>Other<br>*(specify)* ______	1<br>2<br>3<br>4<br>5<br>6<br>-77<br>[ ]	
B16	In the past 12 months, what was the average income wildlife trading brought to you (xxx thousands VND/month)?	Xxxx<br>No income from wildlife farming yet<br>Unknown	 0<br>-99	 0→B19<br>-99→B13
B17	How much does wildlife farming contribute to your total annual household income in the past 12 months?	%<br>No income from wildlife farming yet<br>Unknown	[ ]<br>0<br>-99	
B18	To what extent has this contribution rate increased, decreased or remained the same over the past 12 months?	Increased<br>Decreased<br>Unchanged<br>Unknown	1<br>2<br>3<br>-99	
B19	Besides wildlife farming, are you involved in any other wildlife-related activities?<br>*Interviewer:*<br>• *Read options*<br>• *Select ALL that apply*	Hunting/trapping<br>Slaughtering wild animals<br>Processing wild animal products<br>Trading live wild animals<br>Trading slaughtered wild animals<br>Consuming meat of wild animals<br>Consuming other wildlife products<br>Harvesting bat guano<br>Other<br>*(specify)* ______	1<br>2<br>3<br>4<br>5<br>6<br>7<br>8<br>-77<br>[ ]	

C	WILDLIFE FARMING ACTIVITIES			
C1	Is the farm licensed by government authority?	No<br>Yes<br>Do not know	0<br>1<br>-99	
C2	During the process of wildlife farming, do you keep records of any of the following information?<br>*Interviewer:*	In and out<br>Feed origin<br>Diseases and treatment	1<br>2<br>3	

	• *Read options*<br>• *Select ALL that apply*	Vaccination<br>No records<br>Other<br>*(specify)* ______	4<br>0<br>-77<br>[ ]	
C3	Have you participated in training or receive guidance on wildlife farming procedures, veterinary care, and environmental protection related to wildlife farming?	No<br>Yes	0<br>1	
C4	How many times have authorities inspected your wildlife farm in the past 12 months?	Xxxx times<br>Do not remember<br>Do not know	[ ]<br>-88<br>-99	

C5. Information about wildlife species currently raised in the farm

				The most recent time of **importing** wildlife						
C5.1 Wildlife species raised at your farm<br><br>**Droplist**<br>1. Bats<br>2. Bamboo rats<br>3. Asian Palm Civets<br>4. Masked Palm Civets<br>5. Wild boars<br>6. Snacks<br>7. Hedgehogs<br>8. Pythons<br>9. Crocodiles<br>10. Other *(specify)* ______	C5.2 Number of [SPECIES] currently raised	C5.3 When did you start raising [SPECIES]?<br><br>*(yyyy)*	C5.4 What is the purpose of raising [SPECIES] *(select all that apply)*<br><br>1. For meat at home<br>2. For meat to sell<br>3. Breeding<br>4. Medicine<br>5. Pet<br>6. Other *(specify)* ______	C5.5 When was the most recent introduction of new [SPECIES] in your farm?<br><br>*(mm/yyyy)*	C5.6 What was the number of individuals of [SPECIES] most recently introduced to your farm? How many kilograms in total?<br>…individuals<br>…kg<br><br>*NPV: please convert to kg*	C5.7 Which sources did you purchase [SPECIES] from?<br><br>1. Self-breeding<br>2. Purchase from a single farm<br>3. Purchase from various farms<br>4. Purchase from wholesale market<br>5. Purchase from local hunters<br>6. Other *(specify)*____	C5.8 How much did you have to pay for [SPECIES] per kg (VND/kg)<br><br>*NPV: please convert to VND/kg*	C5.9 In the most recent import of new breeder animals of [SPECIES] , which information did you check? *(select all that apply)*<br>1. Do not check<br>2. Health<br>3. Origin<br>4. Price<br>5. Other *(specify)* ______	C5.10 How were [SPECIES] transported to your farm? (*select all that apply)*<br><br>1. Transported by myself on a motorcycle/bicycle<br>2. Transported by myself by car/pickup truck/tricycle<br>3. Seller delivers to my location by motorcycle/bicycle<br>4. Seller delivers to my location by car/pickup truck/tricycle<br>5. Other (specify) ______	C5.11 Where did newly purchased wild animals usually kept?<br>1. In a separate isolation area<br>2. In separate cages but in the same vicinity as other animals<br>3. Together in the same area with other animals<br>4. Other (specify) ______
**1.**										
**2.**										
**3.**										
**4.**										
**5.**										

The most recent time of **raising** wildlife				The most recent time of **trading**							
C5.12 Which kinds of feed do you usually use to raise [SPECIES]?<br>*NPV: Select ALL that apply*<br>1. Commercial feed<br>2. Uncooked mixed grains /feed<br>3. Self-cooked grains /porridge /food<br>4. Homegrown fruits and vegetables<br>5. Fruits and vegetables purchased from the market/traders<br>6. Other (specify) _____	C5.13 What is the main source of drinking water for [SPECIES]?<br>1. Tap water<br>2. Drilled well water/ dug well water<br>3. Rainwater<br>4. Pond/ Lake/ River<br>5. Stream/ Creek<br>6. Other (specify) _____	C5.14 Do you give [SPECIES] antibiotics in the following circumstances?<br>*Select all that apply*<br>1. Animals showing anorexia<br>2. Weather changes<br>3. Animals having diarrhea<br>4. Animals showing symptoms like coughing, rhinorrhea, or sore eyes<br>5. Animals having wounds or injuries<br>6. Other *(specify)* _____	C5.15 How do you give [SPECIES] antibiotics when [ANSWER TO C5.14]?<br>*Select all that apply*<br>1. Mixing into feed<br>2. Mixing/ dissolving in drinking water<br>3. Orally<br>4. Injecting<br>5. Other *(specify)* _____	C5.16 When was the most recent time you sold [SPECIES]?<br>*1. (mm/yyyy)*<br>*2. Not yet*<br>2 >> skip to next animal	C5.17 What was the longest raising period of [SPECIES] until they are ready for sale?<br>*(…months)*	C5.18 Who was the most recent buyer of [SPECIES]?<br>1. Other farm owners<br>2. Traders<br>3. Consumers<br>4. Restaurants<br>5. Other *(specify)* _____	C5.19 What was the trading volume of [SPECIES] (kg) in the most recent trading time?	C5.20 In the most recent trade, what was the selling price per kg of [SPECIES]? (VND/kg)	C5.21 How were [SPECIES] transported from your farm?<br>*Select all that apply*<br>1. Transported by buyers on motorcycle/ bicycle.<br>2. Transported by buyers on a car/pickup truck/three-wheeler.<br>3. Seller delivers to buyer's location using a motorcycle/ bicycle.<br>4. Seller delivers to buyer's location using a car/pickup truck/three-wheeler.<br>*5.* Other *(specify)* _____	C5.23 [SPECIES] you usually sold are still alive or slaughtered?<br>1. Alive<br>2. Slaughtered<br>3. Both

C6. In the next year, including the animals you are currently raising, do you plan to raise any of the following wildlife species, and what is the reason for the change?

C6.1 Species<br><br>**(drop list)**<br>1. Bats<br>2. Bamboo rats<br>3. Asian palm civets<br>4. Masked palm civets<br>5. Wild boars/Native pigs<br>6. Snakes<br>7. Porcupines<br>8. Pythons<br>9. Crocodiles<br>10. Other *(specify)*	C6.1 How will [SPECIES] change?<br>1. Increase<br>2. Decrease<br>3. Remain unchanged<br>4. Do not have any plan yet	C6.2 Main reason for the change of [SPECIES]<br>1. Business model change<br>2. State and local regulations<br>3. Consumer demand change<br>4. Diseases<br>5. Interest rates<br>6. Other *(specify)* _____
CODE	CODE	CODE
1		
2		

C7	In the past 5 years, were there any wildlife species that you raised in the past but currently stopped?	No<br>Yes	0<br>1	0 → C10
C8	Which species?<br>*Interviewer:*<br>• *Select ALL that apply*	Bats<br>Bamboo rats<br>Asian palm civets<br>Masked palm civets<br>Wild boars<br>Other<br>*(specify)* ______	1<br>2<br>3<br>4<br>5<br>-77<br>[ ]	
C9	What are the main reasons to stop raising?	Changing business model<br>Government regulations<br>Changing customer preferences<br>Diseases<br>Interest rates<br>Other<br>*(specify)* ______	1<br>2<br>3<br>4<br>5<br>-77<br>[ ]	

C10. Ask about ALL the members involved in the wildlife farming activities at your family's farm in the past 12 months.

	General questions								People currently involved in wildlife farming activities					
**C10.** C10. In the past 12 months, how many people were involved in the main activity of raising wild animals on the farm?<br>1. Respondent himself/her self – person 1<br>2. Person 2<br>3. Person 3<br>4. Person 4<br>5. Person 5<br>6. Person 6<br>7. Person 7<br>8. Person 8<br>9. Person 9	**C10.1 Name**<br>(Those who have been and are currently participating in wildlife farming activities in the past 12 months)	**C10.2 Relationship with head of the household**<br>1. Head of household<br>2. Spouse<br>3. Biological child<br>4. Siblings<br>5. Daughter-in-law/son-in-law<br>6. Parents<br>7. Grandparents<br>8. Niece/nephew<br>9. Hired laborer<br>10. Other *(specify)*	**C10.3 Gender**<br>1. Male<br>2. Female	**C10.4 Year of birth**<br>*yyyy*	**C10.5 Which of the following activities does [NAME] participate in?**<br>*(SHOW CARD)*<br>1. Purchasing breeder animals<br>2. Breeding<br>3. Preparing feed and water<br>4. Feeding and providing water to the animals<br>5. Cleaning the farm/pen<br>6. Purchasing medications<br>7. Treating animals (administering medication)<br>8. Medication injections<br>9. Slaughtering<br>10. Cooking and processing wildlife meat<br>11. Selling products made from wild animals<br>12. Contacting a veterinarian<br>13. Contacting a trader/buyer<br>14. Selling breeder animals<br>15. Selling mature wild animals<br>16. Registering with the local authorities<br>17. Transporting wild animals<br>18. Other (specify)	**C10.6 How many days per week does [NAME] participate in wildlife farming activities?**<br>… days	**C10.7 How many hours per day does [NAME] participate in wildlife farming activities?**<br>*(round to half an hour)*<br>*… hours*	**C10.8 Is [NAME] still involved in wildlife farming activities?**<br>1. Yes<br>2 No<br>(If no skip to C10.14)	**C10.9 Was [NAME] involved in making decision when the family started/ switched to wildlife farming?**<br>1. Very few or no decision<br>2. Some decisions<br>3. Most or all decisions<br>4. NOT APPLICABLE/ NOT SUITABLE	**C10.10 How is [NAME] involved in making decision when the family started/ switched to wildlife farming?**<br>1. Very few or no decision<br>2. Some decisions<br>3. Most or all decisions<br>4. NOT APPLICABLE/ NOT SUITABLE	**C10.11 Is [NAME] involved in making decision regarding treating wildlife diseases?**<br>1. Very few or no decision<br>2. Some decisions<br>3. Most or all decisions<br>4. NOT APPLICABLE/ NOT SUITABLE	**C10.12 Is [NAME] involved in making decision to sell wild animals?**<br>1. Very few or no decision<br>2. Some decisions<br>3. Most or all decisions<br>4. NOT APPLICABLE/ NEVER SOLD BEFORE	**C10.13 How is [NAME] involved in making decision about income earned from wildlife farming activities?**<br>1. Very few or no decision<br>2. Some decisions<br>3. Most or all decisions<br>4. NOT APPLICABLE/ NEVER SOLD BEFORE	**C10.14 Does [NAME] have any type of insurances?**<br>1. Health insurance<br>2. Life insurance<br>3. Social insurance<br>4. Other *(specify)*
		CODE	CODE	NUMBER	CODE	NUMBER	NUMBER		CODE	CODE	CODE	CODE	CODE	CODE
1.														
2.														

D	KNOWLEDGE, ATTITUDE, AND PRACTICE IN PREVENTING ZOONOTIC DISEASES TRANSMISSION			
**NO.**	**QUESTION**	**RESPONSE**	**OPT**	**SKIP**
	**Knowledge**			
D1	Have you ever heard about zoonosis?	No<br>Yes	0<br>1	0 →D3
D2	Can you list the name of some zoonotic diseases in wild animals that you know?<br><br>*Interviewer:*<br>• *Select ALL that apply*	COVID-19<br>SARS<br>MERS<br>Ebola<br>Rabies<br>Avian Influenza<br>*Streptococcus suis* infection<br>Cysticercosis<br>Leptospirosis<br>Other<br>*(specify)* ______	1<br>2<br>3<br>4<br>5<br>6<br>7<br>8<br>9<br>-77<br>[ ]	
D3	In your opinion, can many diseases in wildlife be transmitted to humans	False<br>True<br>Do not know/Uncertain		
D4	In your opinion, which wildlife species can carry pathogens that can be transmitted to humans?<br>*Interviewer:*<br>• *Read options*<br>• *Select ALL that apply*	Bats<br>Bamboo rats<br>Asian palm civets<br>Masked palm civets<br>Wild boars<br>Other<br>*(specify)*______<br>Do not know/Uncertain	1<br>2<br>3<br>4<br>5<br>-77<br>[ ]<br>-99	
D5	Name some **symptoms** in wild animals that can be related to zoonotic diseases.<br><br>*Interviewer:*<br>• *Select ALL that apply*	Fever<br>Diarrhea<br>Coughing<br>Vomiting<br>Anorexia<br>Lethargy<br>Other<br>*(specify)* ______<br>Do not know/Uncertain	1<br>2<br>3<br>4<br>5<br>6<br>-77<br>[ ]<br>-99	
D6	Can diseases in animals be transmitted to humans through **MULTIPLE** transmission pathways?	False<br>True<br>Do not know/Uncertain	0<br>1<br>-99	
D7	Can zoonotic diseases be transmitted by consuming raw or undercooked wildlife meat or products?	False<br>True<br>Do not know/Uncertain	0<br>1<br>-99	
D8	Can zoonotic diseases be transmitted by close contact with sick or dead wildlife?	False<br>True<br>Do not know/Uncertain	0<br>1<br>-99	
D9	Can zoonotic diseases be contracted from an environment contaminated with the excretion of wild animals	False<br>True<br>Do not know/Uncertain	0<br>1<br>-99	
D10	Can zoonotic diseases be transmitted through bites or scratches from wildlife?	False<br>True	0<br>1	

		Do not know/Uncertain	-99	
D11	Can zoonotic diseases be prevented?	False<br>True<br>Do not know/Uncertain	0<br>1<br>-99	
D12	Isolating newly introduced or sick wild animals in separate areas can help prevent the spread of disease on the farm?	False<br>True<br>Do not know/Uncertain	0<br>1<br>-99	
D13	Wash/Sanitizing hands before and after contact with wildlife can reduce the risk of contracting zoonotic diseases?	False<br>True<br>Do not know/Uncertain	0<br>1<br>-99	
D14	Avoiding contact with wildlife while having open wounds can reduce the risk of contracting zoonotic diseases?	False<br>True<br>Do not know/Uncertain	0<br>1<br>-99	
D15	Wearing a face mask when in contact with wildlife can mitigate the risk of contracting zoonotic diseases	False<br>True<br>Do not know/Uncertain	0<br>1<br>-99	
D16	Wearing protective clothing and gloves when in contact with wildlife can reduce the risk of contracting zoonotic diseases?	False<br>True<br>Do not know/Uncertain	0<br>1<br>-99	
	**Attitude**<br>Do you agree with the following statements? *(using showcard)*			
D17	Disease outbreaks can happen in wild animals	Strongly disagree<br>Disagree<br>Neither agree nor disagree<br>Agree<br>Strongly agree	1<br>2<br>3<br>4<br>5	
D18	Vaccination can help prevent some diseases in wild animals	Strongly disagree<br>Disagree<br>Neither agree nor disagree<br>Agree<br>Strongly agree	1<br>2<br>3<br>4<br>5	
D19	Isolating newly introduced or sick wild animals is crucial to prevent the spread of disease on the farm	Strongly disagree<br>Disagree<br>Neither agree nor disagree<br>Agree<br>Strongly agree	1<br>2<br>3<br>4<br>5	
D20	It is necessary to promptly report to veterinary and forestry authorities if a disease outbreak occurs in the wildlife population	Strongly disagree<br>Disagree<br>Neither agree nor disagree<br>Agree<br>Strongly agree	1<br>2<br>3<br>4<br>5	
D21	Some diseases in wildlife can be transmissible and can pose a risk to human health	Strongly disagree<br>Disagree<br>Neither agree nor disagree<br>Agree<br>Strongly agree	1<br>2<br>3<br>4<br>5	
D22	Not using personal protective equipment such as masks, gloves, and protective clothing when in contact with wildlife can increase the risk of	Strongly disagree<br>Disagree<br>Neither agree nor disagree<br>Agree	1<br>2<br>3<br>4	

	contracting zoonotic diseases for me and my family	Strongly agree	5	
D23	I need to promptly report to relevant authorities or healthcare facilities if I or any members of my family show suspected symptoms of zoonotic diseases.	Strongly disagree<br>Disagree<br>Neither agree nor disagree<br>Agree<br>Strongly agree	1<br>2<br>3<br>4<br>5	
D24	Managing and preventing zoonotic disease transmission is the responsibility of the government and healthcare institutions, not my responsibility	Strongly disagree<br>Disagree<br>Neither agree nor disagree<br>Agree<br>Strongly agree	1<br>2<br>3<br>4<br>5	
D25	Consuming abnormal or sick wild animals can increase the risk of contracting zoonotic diseases to me and my family	Strongly disagree<br>Disagree<br>Neither agree nor disagree<br>Agree<br>Strongly agree	1<br>2<br>3<br>4<br>5	
D26	Eating dishes made from undercooked, raw, or partially cooked wildlife meat and products can increase the risk of contracting zoonotic diseases for me and my family.	Strongly disagree<br>Disagree<br>Neither agree nor disagree<br>Agree<br>Strongly agree	1<br>2<br>3<br>4<br>5	
	**Practice**			
D27	Do you check the health information of wild animals that are newly introduced to your farm?	Never<br>Occasionally<br>Always	0<br>1<br>2	
D28	Do you regularly clean the housing area of wild animals in your farm?	Everyday<br>1-2 times/week<br>1-2 times/month<br>1-2 times/quarter<br>1-2 times/year<br>Never<br>Other<br>*(specify)* ______	1<br>2<br>3<br>4<br>5<br>0<br>-77<br>[ ]	
D29	Do you regularly disinfect the housing area of wild animals in your farm?	Everyday<br>1-2 times/ week<br>1-2 times/ month<br>1-2 times/quarter<br>1-2 times/year<br>Never<br>Other<br>*(specify)* ______	1<br>2<br>3<br>4<br>5<br>0<br>-77<br>[ ]	
D30	How is organic (solid) waste from livestock usually treated?<br><br>*Interviewer:*<br>*Select ALL that apply*	Used as fertilizer for plants/for sell<br>Processed in a biogas system<br>Directly used as fertilizer for plants in the garden<br>Sold/given to collectors<br>Disposed in waste disposal areas/public waste collection	1<br>2<br>3<br>4<br>5	

		Buried/burned<br>Other<br>(Specify) ______	6<br>-77<br>[ ]	
D31	How is inorganic (solid) waste from livestock usually treated?<br><br>*Interviewer:*<br>*Select ALL that apply*	Used as fertilizer for plants/for sell<br>Processed in a biogas system<br>Directly used as fertilizer for plants in the garden<br>Sold/given to collectors<br>Disposed in waste disposal areas/public waste collection<br>Buried/burned<br>Other<br>(Specify) ______	1<br>2<br>3<br>4<br>5<br>6<br>-77<br>[ ]	
D32	How is wastewater from the farm treated?<br><br>*Interviewer:*<br>*Select ALL that apply*	Processed in a biogas system<br>Discharged into the public wastewater system<br>Released into the farm's garden<br>Released into the farm's ponds/lakes<br>Not applicable<br>Other<br>*(specify)* ______	1<br>2<br>3<br>4<br>-99<br>-77<br>[ ]	
D33	Do you wash your hands with soap or sanitize them before coming into contact with wild animals?	Never<br>Occasionally<br>Always	0<br>1<br>2	
D34	Do you wash your hands with soap or sanitize them after coming into contact with wild animals?	Never<br>Occasionally<br>Always	0<br>1<br>2	
D35	Do you wear face mask when in contact with wild animals?	Never<br>Occasionally<br>Always	0<br>1<br>2	
D36	Do you wear protective clothing when cleaning the farm and contacting with wild animals?	Never<br>Occasionally<br>Always	0<br>1<br>2	
D37	Do you wear protective glove when cleaning the farm and contacting with wild animals?	Never<br>Occasionally<br>Always	0<br>1<br>2	
D38	Do you isolate sick or abnormal wild animals in a separate area?	Never<br>Occasionally<br>Always	0<br>1<br>2	
D39	Do you seek for vet care service or call a vet when wild animals in your farm are sick or abnormal?	Never<br>Occasionally<br>Always	0<br>1<br>2	
D40	Do you report to the authorities when wild animals in your farms are sick or abnormal?	Never<br>Occasionally<br>Always	0<br>1<br>2	
D41	Do you clean and disinfect the entire farm when wild animals in your farm are sick or abnormal?	Never<br>Occasionally<br>Always	0<br>1<br>2	

E	CONSUMPTION AND COLLECTION OF WILDLIFE PRODUCTS<br>*Appear if B13=6 or 7*			
**NO.**	**QUESTION**	**RESPONSE**	**OPT**	**SKIP**
E1	When was the last time you consumed wildlife meat and other products?	Never ever consumed<br>mm/yyyy	0<br>[ ]	0 → E5
E2	When was the last time you collected wild animal products?	Never ever collected<br>mm/yyyy	0<br>[ ]	
E3	How did you consume wildlife product in your first time?<br>*Interviewer:*<br>• *Read options*<br>• *Select ALL that apply.*	Using in family meals<br>Invited by friends/colleagues<br>Bought it yourself to eat/use<br>Prescribed/advised as medications or suppliments<br>Other<br>*(specify)* ______	1<br>2<br>3<br>4<br>-77<br>[ ]	

E4. The wildlife species that you consume, including animals/products produced on your **farm in the last 12 months** *(list all wildlife species and provide detailed information about the top four most consumed species).*<br>1. Bats<br>2. Bamboo rats<br>3. Asian palm civets<br>4. Masked palm civets<br>5. Wild boars/Native pigs	E4.1 Purpose of using [SPECIES]? *(select all applicable answers)*<br>1. To eat raw meat<br>2. To eat cooked meat<br>3. To eat raw blood pudding<br>4. To eat organs<br>5. For medicinal purposes<br>6. For display<br>7. Other *(specify*	E4.2 When was the most recent time you consumed [SPECIES]?<br>mm/yyyy<br>*NPV: write -99 or -9999 if they do not remember*	E4.3 With whom did you most recently consume [SPECIES]<br>1. Family<br>2. Friends/Colleagues<br>3. Others *(specify)*	E4.4 How often do you consume products from [SPECIES]? *(select all applicable answers)*<br>1. Everyday<br>2. Every week<br>3. Every month<br>4. Once per year<br>5. Several times per year<br>6. Other *(specify)*	E4.5 Where do you purchase [SPECIES] for consumption? *(select all applicable answers)*<br>1. Home farm<br>2. Other farm<br>3. Self-hunting<br>4. Other hunter<br>5. Market<br>6. Middleman<br>7. Restaurant<br>8. Other *(specify*	E4.6 The condition of [SPECIES] at the time of purchase?<br>1. Alive and healthy<br>2. Alive with abnormalities<br>3. Freshly slaughtered<br>4. Slaughtered for some time, stored in cool place<br>5. Other *(specify)*	E4. 7 What is the average amount you consump [SPECIES] in one time (a meal) (g) most recently?<br>*NPV: please convert to g*	E4.8 What is the average purchase price of [SPECIES] per kg/individual<br>*NPV: Please convert to VND/kg*	E4.9 How do you usually purchase [SPECIES]?<br>1. Online<br>2. In person<br>3. Not applicable/at farm<br>4. Other (specify))	E4.10 How do you transport [SPECIES] in the most recent time?<br>1. Transport it myself on a motorcycle/bicycle<br>2. Transport it myself in a car/pickup truck/three-wheeler<br>3. Seller delivers it to my location on a motorcycle/bicycle<br>4. Seller delivers it to my location in a car/pickup truck/three-wheeler<br>5. Not applicable/at farm<br>6. Other *(specify)*	E4.11 In term of raising [SPECIES]… have you ever? *(select all applicable answers)*<br>1. Touched or caught [SPECIES]<br>2. Collected products from [SPECIES]<br>3. Transported [SPECIES]<br>4. Slaughtered [SPECIES]<br>5. Cooked and processed [SPECIES]<br>6. Consumed [SPECIES]<br>7. Other (specify)	E4.12 Did you have any open wounds or cut on your hand when [ANSWER TO E4.10]?<br>1. No<br>2. Yes<br>3/ Do not remember/know	E4.13 Did you get bitten or scratched by [SPECIES] when [ANSWER TO E4.10]?<br>1. No<br>2. Yes<br>3/ Do not remember/know	E4.14 Which protective equipment do you use when [ANSWER FOR E4.10] *(select all applicable answers)*<br>1. Face mask<br>2. Gloves<br>3. Boots/shoes<br>4. Protective clothing<br>5. I don't use any equipment<br>6. Other *(specify)*

6. Others *(specify)*														
	CODE	CODE	CODE	CODE	CODE		NUM BER	NUM BER	COD E	CODE	CODE	CODE	CODE	CODE
1.														
2.														
3.														
4.														
	5. Other wildlife species													

F	MEDICAL HISTORY SECTION			
**NO.**	**QUESTION**	**RESPONSE**	**OPT**	**SKIP**
F1	In the past 12 months, did you experience illness or health issues?	No<br>Yes	0<br>1	0→F4
F2	During those instances, when you were most seriously ill, did you experience any of the following symptoms?<br><br>*Interviewer:*<br>• *Only read the symptoms*<br>• *Select ALL that apply.*<br>•	Fever<br>Headache<br>Fatigue<br>Rash<br>Bruises<br>Nosebleeds<br>Cough<br>Shortness of breath<br>Difficulty breathing<br>Weakness<br>Vomiting<br>Diarrhea<br>Muscle pain<br>Skin ulcers<br>Other<br>*(specify)* ______<br>No specific symptoms	1<br>2<br>3<br>4<br>5<br>6<br>7<br>8<br>9<br>10<br>11<br>12<br>13<br>14<br>-77<br>[ ]<br>0	0 →F4
F3	What did you do when you had any of the above symptoms?	Self treated<br>Traditional healer using oriental medicine<br>Treated at the hospital/health facility<br>Nothing<br>Other<br>*(specify)*___________	1<br>2<br>3<br>0<br>-77<br>[ ]	
F4	In the past 12 months, did any of your family members experience illness or health issues?	No<br>Yes	0<br>1	0 →G1
F5	If yes, what were the symptoms?<br>*Interviewer:*<br>• *Only read the symptoms*<br>• *Select ALL that apply.*	Fever<br>Headache<br>Fatigue<br>Rash<br>Bruises<br>Nosebleeds<br>Cough<br>Shortness of breath<br>Difficulty breathing<br>Weakness<br>Vomiting<br>Diarrhea<br>Muscle pain<br>Skin ulcers<br>Other<br>*(specify)* ______<br>No specific symptoms	1<br>2<br>3<br>4<br>5<br>6<br>7<br>8<br>9<br>10<br>11<br>12<br>13<br>14<br>-77<br>[ ]<br>0	0 →G1
F6	What did your family do when family members had the following symtoms?	Self treated<br>Traditional healer using oriental medicine<br>Treated at the hospital/health facility	1<br>2<br>3	

		Nothing<br>Other<br>*(specify)*__________	0<br>-77<br>[ ]	

G	POLICIES AND WRAP-UP			
**NO.**	**QUESTION**	**RESPONSE**	**OPT**	**SKIP**
G1	Do you know any regulations/policies related to wild animals' business in Vietnam	No<br>Yes	0<br>1	
G2	Do you know any regulations/policies related to zoonotic diseases in Vietnam	No<br>Yes	0<br>1	0 → G5
G3	What are they?<br><br>*Interviewer:*<br>• *Only read the symptoms*	Prohibited to raise wildlife species<br>Conditionally permitted to raise wildlife species<br>Management, transportation, and trading of wildlife<br>Hygiene in animal husbandry, slaughter, transportation, and destruction<br>Zoonotic diseases<br>Other<br>(specify) _____	1<br>2<br>3<br>4<br>5<br>-77<br>[ ]	
G4	What do you think about the application of the available regulations/policies in managing wildlife business and/or zoonotic diseases in Vietnam?	Very easy to follow<br>Easy to follow<br>Neither easy nor difficult<br>Difficult to follow<br>Very difficult to follow	1<br>2<br>3<br>4<br>5	
G5	Which of the following ways are effective in raising awareness among people about **regulations/policies** related to wildlife business and/or zoonotic diseases?<br><br>*Interviewer:*<br>• *Read options*<br>• *Select ALL that apply.*<br>• *Suggestion: Anything else?*	Social media platforms (e.g. Facebook, Tiktok, etc.)<br>Educational workshops and seminars<br>Relevant movies or documentaries<br>Public posters and billboards<br>Specialized TV channels<br>Radio broadcasts<br>Other<br>*(specify)* _____	1<br>2<br>3<br>4<br>5<br>6<br>-77<br>[ ]	
G6	Which of the following are effective ways to **enhance knowledge** about wildlife farming (biosecurity, disease prevention, productivity)?<br>*Interviewer:*<br>• *Read options*<br>• *Select ALL that apply.*<br>• *Suggestion: Anything else?*	Social media platforms (e.g. Facebook, Tiktok, etc.)<br>Educational workshops and seminars<br>Relevant videos or documentaries<br>Public posters and billboards<br>Leaflets<br>Television advertisements<br>Practical handbooks<br>Radio broadcasts<br>Other<br>*(specify)* _____	1<br>2<br>3<br>4<br>5<br>6<br>7<br>8<br>-77<br>[ ]	
G7	Primary phone number for recontact	*(Type in: Numberic only)*<br>Not available	[ ]<br>-99	
G8	Primary phone owner name	_______________		

G9	*(Automatic)* End Timestamp	*(Automatically generated)*		
G10	***[Do not ask]*** GPS location	Lat<br>Long<br>Accuracy	[ ]<br>[ ]<br>[ ]	
G11	***[Do not ask]*** Interviewer's comments about the interview	*(Type in)*	[ ]	

Thank you very much!
