# Supplementary material for: Seroprevalence and risk factors of hantavirus and hepatitis E virus exposure among wildlife farmers in Vietnam
Source: PLoS One. 2025 Aug 7;20(8):e0329570. doi: 10.1371/journal.pone.0329570 (PMC12331095; doi:10.1371/journal.pone.0329570)
Supplement: S2 Table — (PDF) [file pone.0329570.s002.pdf]

S2 Table. Serological results among study participants by demographic characteristics and reported activities.

Variable	Any seropositivity	Hantavirus	Hepatitis E virus
**Total** (n = 207)	70 (33.8%)	21 (10.1%)	55 (26.6%)
**Province**			
Lao Cai (n = 46)	23 (50%)	5 (10.9%)	21 (45.7%)
Dong Nai (n = 161)	47 (29.2%)	16 (9.9%)	34 (21.1%)
**Gender**			
Man (n = 130)	50 (38.5%)	15 (11.5%)	41 (31.5%)
Woman (n = 77)	20 (26.0%)	6 (7.8%)	14 (18.2%)
**Age (median)**	59	59	59
**Ethnicity**			
Kinh (n = 168)	48 (28.6%)	18 (10.7%)	34 (20.2%)
Ethnic minority (n = 39)	22 (56.4%)	3 (7.7%)	21 (53.8%)
**Education**			
No formal education (n = 7)	3 (42.9%)	0 (0%)	3 (42.9%)
Primary school (n = 25)	11 (44.0%)	4 (16.0%)	8 (32.0%)
Secondary school (n = 80)	28 (35.0%)	8 (10.0%)	22 (27.5%)
High school (n = 62)	22 (35.5%)	6 (9.7%)	18 (29.0%)
≥College/University (n = 33)	6 (18.2%)	3 (9.1%)	4 (12.1%)
**Additional occupation**			
No other occupation (n = 51)	14 (27.5%)	4 (7.8%)	12 (23.5%)
Government employee (n = 16)	4 (25.0%)	2 (12.5%)	3 (18.8%)
Private company (n = 12)	2 (16.7%)	1 (8.3%)	1 (8.3%)
Trading/ self-employed (n = 53)	18 (34.0%)	3 (5.7%)	15 (28.3%)
Plant farming/crop cultivation (n=51)	23 (45.1%)	7 (13.7%)	18 (35.3%)
Livestock and poultry farming (n = 24)	9 (37.5%)	3 (12.5%)	6 (25.0%)
**Species farmed**			
Bats (n = 16)	7 (43.8%)	2 (12.5%)	5 (31.3%)
Wild boars (n = 53)	20 (37.7%)	3 (5.7%)	19 (35.8%)
Bamboo rats (n = 51)	17 (33.3%)	6 (11.8%)	11 (21.6%)
Civets (n = 87)	26 (29.9%)	10 (11.5%)	20 (30.0%)
**Specific wildlife activities**			
Hunting/trapping (n = 15)	9 (60.0%)	2 (13.3%)	8 (53.3%)
Consuming other products (n = 10)	4 (40.0%)	2 (20.0%)	3 (30.0%)
Slaughtering (n = 51)	20 (39.2%)	5 (9.8%)	17 (13.7%)
Farming wild animals only (n = 49)	18 (36.7%)	9 (18.4%)	12 (24.5%)
Consuming wild meat (n = 129)	43 (33.3%)	10 (7.8%)	36 (27.9%)
Processing (n = 64)	20 (31.3%)	5 (7.8%)	17 (26.6%)
Trading live wild animals (n = 90)	28 (31.1%)	7 (7.8%)	22 (24.4%)

Variable	Any seropositivity	Hantavirus	Hepatitis E virus
Harvesting bat guano (n = 13)	4 (30.8%)	1 (7.7%)	3 (23.1%)
Trading slaughtered wild animals (n = 19)	4 (21.1%)	0 (0%)	4 (21.1%)
**Washing hands with soap or sanitize them before contact with wild animals**			
Never (n = 105)	35 (33.3%)	12 (11.4%)	27 (25.7%)
Sometimes (n = 40)	7 (17.5%)	3 (7.5%)	4 (10.0%)
Always (n = 72)	28 (38.9%)	6 (8.3%)	24 (33.3%)
**Washing hands with soap or sanitize them after contact with wild animals**			
Never (n = 15)	5 (33.3%)	1 (6.7%)	4 (26.7%)
Sometimes (n = 14)	6 (42.9%)	0 (0%)	6 (42.9%)
Always (n = 178)	59 (33.1%)	20 (11.2%)	45 (25.3%)
**Clean wildlife farms regularly**			
Never (n = 14)	6 (42.9%)	1 (7.1%)	5 (35.7%)
Sometimes (n = 68)	20 (29.4%)	5 (7.4%)	16 (23.5%)
Always (n = 125)	44 (35.2%)	15 (12.0%)	34 (27.2%)
**Disinfect wildlife farms regularly**			
Never (n = 145)	49 (33.8%)	11 (7.6%)	40 (27.6%)
Sometimes (n = 60)	20 (33.3%)	9 (15.0%)	15 (25.0%)
Always (n = 2)	1 (50.0%)	1 (50.0%)	0 (0%)
**Processed organic waste from the wildlife farm in a biogas system**			
No (n = 182)	61 (33.5%)	17 (9.3%)	49 (26.9%)
Yes (n = 25)	9 (36.0%)	4 (16.0%)	6 (24.0%)
**Disposed of inorganic waste from the wildlife farm in the waste disposal area**			
No (n = 166)	62 (37.3%)	19 (11.4%)	49 (29.5%)
Yes (n = 41)	8 (19.5%)	2 (4.9%)	6 (14.6%)
**Processed water waste from the wildlife farm in a biogas system**			
No (n = 167)	57 (34.1%)	17 (10.2%)	45 (26.9%)
Yes (n = 40)	13 (32.5%)	4 (10.0%)	10 (25.0%)
**Clean and disinfect the entire farm when wild animals get sick or die**			
Never (n = 73)	27 (36.9%)	6 (8.2%)	23 (31.5%)
Sometimes (n = 15)	3 (20.0%)	0 (0%)	3 (20.0%)
Always (n = 119)	40 (33.6%)	15 (12.6%)	29 (24.4%)
**Wear a face mask when in contact with wild animals**			
Never (n = 68)	22 (32.3%)	7 (10.3%)	17 (25.0%)
Sometimes (n = 59)	20 (33.9%)	7 (11.9%)	15 (25.4%)
Always (n = 80)	28 (35.0%)	7 (8.8%)	23 (28.8%)
**Wear protective clothing when in contact with wild animals**			
Never (n = 170)	57 (33.5%)	19 (11.2%)	43 (25.3%)
Sometimes (n = 16)	4 (25.0%)	1 (6.3%)	3 (18.8%)
Always (n = 21)	9 (42.9%)	1 (4.8%)	9 (42.9%)
**Wear gloves when in contact with wild animals**			
Never (n = 105)	33 (31.4%)	11 (10.5%)	26 (24.8%)
Sometimes (n = 54)	19 (35.2%)	5 (9.3%)	14 (25.9%)
Always (n = 48)	18 (37.5%)	5 (10.4%)	15 (31.3%)
**Raising mixed animals (domestic and wild animals) in the same place**			
No (n = 108)	34 (31.5%)	14 (13.0%)	25 (23.1%)
Yes (n = 99)	36 (36.4%)	7 (7.1%)	30 (30.3%)
**Consume raw meat or raw wild animal products**			
No (n = 196)	62 (31.6%)	20 (10.2%)	48 (24.5%)

Variable	Any seropositivity	Hantavirus	Hepatitis E virus
Yes (n = 11)	8 (72.7%)	1 (9.1%)	7 (63.6%)
**Pets can enter the wild animal places easily**			
No (n = 114)	38 (33.3%)	12 (10.5%)	30 (26.3%)
Yes (n = 93)	32 (34.4%)	9 (9.7%)	25 (26.8%)
**Isolate sick or abnormal wild animals in a separate area**			
Never (n = 64)	23 (35.9%)	6 (9.4%)	18 (28.6%)
Sometimes (n = 11)	3 (27.3%)	1 (9.1%)	3 (24.3%)
Always (n = 132)	44 (33.3%)	14 (10.6%)	34 (25.8%)
**Seek for vet care services or call a vet when wild animals get sick or abnormal**			
Never (n = 126)	43 (34.1%)	10 (7.9%)	36 (28.6%)
Sometimes (n = 37)	12 (32.4%)	5 (13.5%)	9 (24.3%)
Always (n = 44)	15 (34.1%)	6 (13.6%)	10 (22.7%)
**Health check of newly wild animals**			
Never (n = 44)	18 (40.9%)	6 (13.6%)	13 (29.5%)
Sometimes (n = 7)	3 (42.9%)	0 (0%)	3 (42.9%)
Always (n = 156)	49 (31.4%)	15 (9.6%)	39 (25.0%)
**Report to the authorities when wild animals get sick or died**			
Always (n = 29)	10 (34.5%)	2 (6.9%)	9 (31.0%)
Never (n = 156)	53 (34.0%)	16 (10.3%)	42 (26.9%)
Sometimes (n = 22)	7 (31.8%)	3 (4.5%)	4 (27.3%)
**Altitude (m)**			
< 150 (n = 176)	53 (30.1%)	18 (22.2%)	39 (48.1%)
150 – 200 (n = 12)	10 (83.3%)	2 (6.3%)	10 (31.3%)
> 200 (n = 19)	7 (36.8%)	1 (5.3%)	6 (31.6%)
**Mammalian diversity (species)**			
< 100 (n = 81)	25 (30.9%)	8 (9.9%)	20 (24.7%)
100 – 140 (n = 32)	17 (53.1%)	5 (15.6%)	15 (46.9%)
> 140 (n = 94)	28 (29.8%)	8 (8.5%)	20 (21.3%)
