# Supplementary material for: Seroprevalence and risk factors of hantavirus and hepatitis E virus exposure among wildlife farmers in Vietnam
Source: PLoS One. 2025 Aug 7;20(8):e0329570. doi: 10.1371/journal.pone.0329570 (PMC12331095; doi:10.1371/journal.pone.0329570)
Supplement: S3 Table — (PDF) [file pone.0329570.s003.pdf]

S3 Table. Univariable analysis of factors associated with any seropositivity, anti-hantavirus, and anti-HEV.

Variable		Any seropositivity		Hantavirus		Hepatitis E	
		OR (95%CI)	p-value	OR (95%CI)	p-value	OR (95%CI)	p-value
**Gender**							
	Woman	ref		ref		ref	
	Man	1.8 (1.0 – 3.4)	0.07	1.5 (0.6 – 4.5)	0.39	2.1 (1.1 – 4.2)	0.04*
**Age**		1.0 (1.0 – 1.04)	0.19	1.0 (1.0 – 1.03)	0.74	1.0 (1.0 – 1.1)	0.10
**Ethnicity**							
	Kinh	ref					
	Ethnic minority	3.2 (1.5 - 6.7)	<0.01*	0.7 (0.2 – 2.2)	0.58	4.6 (2.2 – 9.7)	<0.01*
**Education**							
	No formal education	ref		0		ref	
	Primary school	1.0 (0.2 – 6.3)	0.96	ref		0.6 (0.1 – 3.8)	0.59
	Secondary school	0.7 (0.1 – 3.9)	0.67	0.6 (0.2 – 2.3)	0.41	0.5 (0.1 – 2.7)	0.39
	High school	0.7 (0.1 – 4.0)	0.70	0.6 (0.1 – 2.4)	0.41	0.5 (0.1 – 3.0)	0.46
	≥College/ University	0.3 (0.1 – 1.8)	0.17	0.6 (0.1 – 2.6)	0.43	0.2 (0.0 – 1.2)	0.07
**Additional occupation**							
	No other occupation	ref		ref		ref	
	Government employee	0.9 (0.2 – 3.0)	0.84	1.7 (0.2 – 9.6)	0.96	0.8 (0.2 – 2.8)	0.69
	Private company	0.5 (0.1 – 2.3)	0.44	1.1 (0.1 – 8.2)	0.57	0.3 (0.0 – 1.8)	0.27
	Trading/ self-employed	1.4 (0.6 – 3.2)	0.47	1.0 (0.2 – 4.3)	0.96	1.3 (0.5 – 3.1)	0.58
	Plant farming/crop cultivation	2.2 (1.0 – 5.0)	0.07	1.9 (0.5 – 7.5)	0.34	1.8 (0.8 – 4.3)	0.19
	Livestock and poultry farming	1.6 (0.6 – 4.4)	0.38	1.7 (0.3 – 8.3)	0.52	1.1 (0.3 – 3.3)	0.89
**Species farmed**							
	Bamboo rats	ref		ref		ref	
	Bats	1.6 (05 – 4.9)	0.45	1.1 (0.1 – 5.3)	0.94	1.7 (0.4 – 5.7)	0.43
	Wild boars	1.2 (0.5 – 2.7)	0.64	0.5 (0.1 – 1.8)	0.28	2.0 (0.9 – 5.0)	0.11
	Civets	0.9 (0.4 – 1.8)	0.67	1.0 (0.3 – 3.0)	0.96	1.1 (0.5 – 2.6)	0.85
**Specific wildlife activities**							
**Hunting/trapping**							
	No	ref		ref		ref	

Variable	Any seropositivity OR (95%CI)	p-value	Hantavirus OR (95%CI)	p-value	Hepatitis E OR (95%CI)	p-value
Yes	3.2 (1.1 – 10)	0.03*	1.4 (0.2 – 5.6)	0.67	3.5 (1.2 – 10.5)	0.02*
**Slaughtering**						
No	ref		ref		ref	
Yes	1.4 (0.7 – 2.6)	0.35	1.0 (0.3 – 2.6)	0.93	1.6 (0.8 – 3.1)	0.21
**Processing**						
No	ref		ref		ref	
Yes	0.8 (0.4 – 1.6)	0.60	0.7 (0.2 – 1.8)	0.46	1.0 (0.5 – 1.9)	0.99
**Trading live wild animals**						
No	ref		ref		ref	
Yes	0.8 (0.4 – 1.4)	0.47	0.6 (0.2 – 1.6)	0.33	0.8 (0.4 – 1.5)	0.54
**Trading slaughtered wild animals**						
No	ref		ref		ref	
Yes	0.5 (0.1 – 1.4)	0.23	0		0.7 (0.2 – 2.1)	0.57
**Consuming wild meat**						
No	ref		ref		ref	
Yes	0.9 (0.5 – 1.7)	0.85	0.5 (0.2 – 1.3)	0.15	1.2 (0.6 – 2.3)	0.58
**Consuming other wildlife products**						
No	ref		ref		ref	
Yes	1.3 (0.3 – 4.8)	0.67	2.3 (0.3 – 10.2)	0.30	1.2 (0.3 – 4.5)	0.80
**Harvesting bat guano**						
No	ref		ref		ref	
Yes	0.9 (0.2 – 2.8)	0.81	0.7 (0.0 – 4.0)	0.76	0.8 (0.2 – 2.8)	0.77
**Farming wild animals only**						
No	ref		ref		ref	
Yes	1.2 (0.6 – 2.3)	0.62	2.7 (1.1 – 6.9)	0.03*	0.9 (0.4 – 1.8)	0.71
**Washing hands with soap or sanitize them before coming into contact with wild animals**						
Never	ref		ref		ref	
Sometimes	0.6 (0.2 – 1.5)	0.30	0.9 (0.2 – 3.0)	0.83	0.4 (0.1 – 1.3)	0.16
Always	1.3 (0.7 – 2.4)	0.45	0.7 (0.2 – 1.9)	0.51	1.4 (0.7 – 2.8)	0.27
**Washing hands with soap or sanitize them after contact with wild animals**						
Never	ref		ref		ref	
Sometimes	1.5 (0.3 – 7.1)	0.60	0		2.1 (0.4 – 10.5)	0.36
Always	1.0 (0.3 – 3.3)	0.99	1.8 (0.3 – 33.0)	0.59	0.9 (0.3 - 3.5)	0.91
**Clean wildlife farms regularly**						
Never	ref		ref		ref	
Sometimes	0.6	0.33	1.0	0.99	0.6	0.35

Variable	Any seropositivity		Hantavirus		Hepatitis E	
	OR (95%CI)	p-value	OR (95%CI)	p-value	OR (95%CI)	p-value
	(0.2 – 1.9)		(0.1 – 20.6)		(0.2 – 2.0)	
Always	0.7 (0.2 – 2.3)	0.57	1.8 (0.3 – 33.3)	0.59	0.7 (0.2 – 2.3)	0.50
**Disinfect wildlife farms regularly**						
Never	ref		ref		ref	
Sometimes	1.0 (0.5 – 1.8)	0.95	2.1 (0.8 – 5.5)	0.1	0.9 (0.4 – 1.7)	0.70
Always	2.0 (0.1 – 50)	0.64	12.2 (0.5 – 322)	0.08	0	
**Processed organic waste from the wildlife farm in a biogas system**						
No	ref		ref		ref	
Yes	1.1 (0.4 – 2.6)	0.81	1.8 (0.5 – 5.6)	0.31	0.9 (0.3 – 2.2)	0.76
**Disposed of inorganic waste from the wildlife farm in the waste disposal area**						
No	ref		ref		ref	
Yes	0.4 (0.2 – 0.9)	0.03*	0.4 (0.1 – 1.4)	0.23	0.4 (0.1 – 1.0)	0.06
**Processed water waste from the wildlife farm in a biogas system**						
No	ref		ref		ref	
Yes	0.9 (0.4 – 1.9)	0.85	1.0 (0.3 – 2.8)	0.97	0.9 (0.4 – 1.9)	0.80
**Clean and disinfect the entire farm when wild animals get sick or die**						
Never	ref		ref		ref	
Sometimes	0.4 (0.1 – 1.5)	0.22	0		0.5 (0.1 – 1.9)	0.38
Always	0.9 (0.5 – 1.6)	0.63	1.6 (0.6 – 4.7)	0.35	0.7 (0.4 – 1.3)	0.28
**Wear a face mask when in contact with wild animals**						
Never	ref		ref		ref	
Sometimes	1.1 (0.5 – 2.3)	0.85	1.2 (0.4 – 3.6)	0.78	1.0 (0.5 – 2.3)	0.96
Always	1.1 (0.6 – 2.2)	0.73	0.8 (0.3 – 2.6)	0.75	1.2 (0.6 – 2.5)	0.61
**Wear protective clothing when in contact with wild animals**						
Never	ref		ref		ref	
Sometimes	0.7 (0.2 – 2.0)	0.49	0.5 (0.0 – 2.9)	0.55	0.7 (0.2 – 2.2)	0.56
Always	1.5 (0.5 – 3.7)	0.40	0.4 (0.0 – 2.1)	0.38	2.2 (0.9 – 5.6)	0.09
**Wear gloves when in contact with wild animals**						
Never	ref		ref		ref	
Sometimes	1.2 (0.6 – 2.4)	0.63	0.9 (0.3 – 2.5)	0.81	1.1 (0.5 – 2.2)	0.87
Always	1.3 (0.6 – 2.7)	0.46	1.0 (0.3 – 2.9)	0.99	1.4 (0.6 – 2.9)	0.40
**Raising mixed animals (domestic and wild animals) in the same place**						
No	ref		ref		ref	
Yes	1.2 (0.7 – 2.2)	0.46	0.5 (0.2 – 1.3)	0.17	1.4 (0.8 – 2.7)	0.25

Variable	Any seropositivity		Hantavirus		Hepatitis E	
	OR (95%CI)	p-value	OR (95%CI)	p-value	OR (95%CI)	p-value
**Consume raw meat or raw wild animal products**						
No	ref		ref		ref	
Yes	5.8 (1.6 – 27)	0.01*	0.9 (0.0 – 5.0)	0.91	5.4 (1.6 – 21.3)	<0.01*
**Pets can enter the wild animal places easily**						
No	ref		ref		ref	
Yes	1.0 (0.6 – 1.9)	0.87	0.9 (0.4 – 2.3)	0.84	1.0 (0.6 – 1.9)	0.93
**Isolate sick or abnormal wild animals in a separate area**						
Never	ref		ref		ref	
Sometimes	0.7 (0.1 – 2.6)	0.58	1.0 (0.1 – 6.6)	0.98	1.0 (0.2 – 3.7)	0.95
Always	0.9 (0.5 – 1.7)	0.72	1.1 (0.4 – 3.4)	0.79	0.9 (0.5 – 1.8)	0.72
**Seek for vet care services or call a vet when wild animals get sick or abnormal**						
Never	ref		ref		ref	
Sometimes	0.9 (0.4 – 2.0)	0.84	1.8 (0.5 – 5.5)	0.31	0.8 (0.3 – 1.8)	0.61
Always	1.0 (0.5 – 2.0)	0.99	1.8 (0.6 – 5.3)	0.27	0.7 (0.3 – 1.6)	0.45
**Health check of newly wild animals**						
Never	ref		ref		ref	
Sometimes	1.1 (0.2 – 5.5)	0.92	0		1.8 (0.3 – 9.3)	0.48
Always	0.7 (0.3 – 1.3)	0.24	0.7 (0.3 – 2.0)	0.45	0.8 (0.3 – 1.7)	0.54
**Report to the authorities when wild animals get sick or die**						
Never	ref		ref		ref	
Sometimes	0.9 (0.3 – 2.3)	0.84	1.4 (0.3 – 4.6)	0.58	0.6 (0.2 – 1.7)	0.39
Always	1.0 (0.4 – 2.3)	0.96	0.6 (0.1 – 2.5)	0.63	1.2 (0.5 – 2.8)	0.65
**Altitude (m)**						
< 150	ref		ref		ref	
150 – 200	1.4 (0.5 – 3.6)	0.54	0.5 (0.0 – 2.5)	0.50	1.6 (0.5 – 4.4)	0.36
> 200	11.6 (2.9 – 77.2)	<0.01*	1.8 (0.3 – 2.6)	0.49	17.6 (4.4 – 117.4)	<0.01*
**Mammalian diversity (species)**	1.0 (1.0 – 1.0)	0.03	1.0 (1.0 – 1.0)	0.53	1.0 (1.0 – 1.0)	0.01
< 100	ref		ref		ref	
100 – 140	1.0 (0.5 – 1.8)	0.88	0.8 (0.3 – 2.4)	0.76	0.8 (0.4 – 1.7)	0.59
> 140	2.5 (1.1 – 5.9)	0.03*	1.7 (0.5 – 5.5)	0.39	2.7 (1.1 – 6.4)	0.02*

* Significant factors at p ≤ 0.05

ref: Reference category

OR: odds ratio; CI: confidence interval
