# Supplementary material for: Seroprevalence and risk factors of hantavirus and hepatitis E virus exposure among wildlife farmers in Vietnam
Source: PLoS One. 2025 Aug 7;20(8):e0329570. doi: 10.1371/journal.pone.0329570 (PMC12331095; doi:10.1371/journal.pone.0329570)
Supplement: Attachment — Submitted filename: Response to Editors and Reviewers.docx [file pone.0329570.s004.docx]

Dear Editors and Reviewers,

We appreciate the opportunity to revise and resubmit our manuscript entitled “Seroprevalence and risk factors of hantavirus and hepatitis E virus exposure among wildlife farmers in Vietnam" (Manuscript ID: PONE-D-25-15935). We thank the reviewers and editorial team for their constructive comments, which have helped us improve the quality and clarity of our work.

Please find below our responses to the additional editorial requirements and reviewers' comments:

| **Journal Requirements** |  |
| --- | --- |
| **Comments** | **Responses** |
| Please ensure that your manuscript meets PLOS ONE's style requirements, including those for file naming. | We have revised our manuscript to comply with PLOS ONE formatting guidelines, including those for file naming. |
| Please include a complete copy of PLOS’ questionnaire on inclusivity in global research in your revised manuscript | We acknowledge the journal’s policy on transparency in global research practices. The questionnaire used in our study has already been published and cited within the manuscript (ref. 62 – line 209). However, to comply with PLOS ONE’s editorial requirements, we have also uploaded a copy of the questionnaire as a Supporting Information file in this submission. The file is named: S1_File_Questionnaire.pdf |
| Role of funders | Please help to revise the statement as follows: “This work was part of the CGIAR initiative on One Health, which was supported by contributors to the CGIAR Trust Fund (<https://www.cgiar.org/funders>). In addition, JFL’s time was funded by FORMAS (2021–00833), HSL's time was supported by the National Research Foundation of Korea (NRF) grant funded by the Korea government (MSIT) (RS-2022-NR068754). The funders had no role in study design, data collection and analysis, decision to publish, or preparation of the manuscript.” |
| Figures 1 and 3 – Map copyright and licensing | Figures 1 and 3 were created by the authors using QGIS software, based on open-source administrative boundary data obtained from the Humanitarian Data Exchange (HDX): <https://data.humdata.org/dataset/cod-ab-vnm>. The data are licensed under the Creative Commons Attribution 3.0 IGO (CC BY-IGO) license.  Following PLOS ONE’s copyright requirements, we revised the captions for figs 1 and 3 to include this information:  Line 152 – 155: **Fig 1. Map of Vietnam and locations of the two provinces selected for the study.**  Source of Vietnam administrative boundary data: <https://data.humdata.org/dataset/cod-ab-vnm>, accessed March 01, 2025, licensed under [CC-BY-IGO](https://creativecommons.org/licenses/by/3.0/igo/).  Line 350 – 356: **Fig** **3**. **Location of 77 serologically positive samples in study districts (yellow) in two provinces.** The map visually emphasizes the spatial distribution of these seropositive individuals, with markers representing their locations within these regions: red dots for hantavirus IgG, orange dots for HEV IgG, and yellow stars for hantavirus IgM (observed only in Dong Nai). Source of Vietnam administrative boundary data: <https://data.humdata.org/dataset/cod-ab-vnm>, accessed March 01, 2025, licensed under [CC-BY-IGO](https://creativecommons.org/licenses/by/3.0/igo/). |
| **Reviewers' comments to the Authors** |  |
| **Comments** | **Responses** |
| **Reviewer #1** |  |
| A major aim of the study is to assess risk factors for hantavirus and HEV, but the introduction contains minimal discussion of what we know about risk factors for these specific viruses from prior studies in other settings. The paper would be strengthened if this prior work were discussed in the introduction and used to motivate the key variables that the authors include in their analysis: age, gender, geography, and behaviors that lead people to interact more closely with wildlife. The authors provide a rich discussion of these risk factors in the discussion and could use some of these details as background and motivation for the analysis. | We thank the reviewer for this insightful suggestion. We have moved some information from the Discussion section and revised the Introduction to provide more background for both hantavirus and hepatitis E virus (HEV) (line 66 – 72 and line 74 – 95). We have also added information to the Introduction to better frame the rationale for our variable selection (i.e., age, gender, geography, and behavioural interactions with wildlife) based on established literature.  Line 100 – 103: *“Research in both endemic and non-endemic areas has revealed demographic and behavioural risk factors associated with hantavirus and HEV infections. Hantavirus risk is linked to exposure to rodent excreta [53], while HEV is associated with older age, man gender, poor sanitation, undercooked meat, and animal contact in farming settings [32,33,54–56].”* |
| Backward selection can often lead to false positives (Type I errors) and can also fail to identify important predictors that might be excluded during the selection process. I suggest the authors consider fitting their model with all the predictor variables they think important for understanding infection patterns and base their inference on that complete model. This paper provides more detail on issues with stepwise model fitting:  Mundry, R., & Nunn, C. L. (2009). Stepwise model fitting and statistical inference: turning noise into signal pollution. The American Naturalist, 173(1), 119-123. | We appreciate the reviewer's feedback regarding the use of backward selection and acknowledge the potential for Type I errors and the exclusion of important predictors. We agree that a fully saturated model is a valid approach and carefully considered this during our analysis. Our decision to employ backward selection was guided by several key considerations, particularly given the exploratory nature of this study, which is among the first to investigate hantavirus and HEV seroprevalence in wildlife farmers in Vietnam.  As Thayer (2002) noted, in early-stage or exploratory studies, stepwise methods can be valuable for "variable evaluation" and for developing "a theory or models that can be tested with further research." This aligns with our aim to identify potential associations worth exploring in future research. Moreover, Dohoo et al. (2010) pointed out that including too many predictors in a full model increases the risk of multicollinearity and the likelihood of retaining variables that may appear significant due to chance but are not truly relevant. They emphasized that sometimes, it is necessary to reduce the number of variables considered during model building. Similarly, Chowdhury and Turin (2020) also highlighted the advantages of backward elimination in exploratory analysis. They noted that backward stepwise regression helps refine the model by eliminating unnecessary variables, thereby improving interpretability and model parsimony.  In our analysis, we began with a full model including all theoretically relevant variables and interactions, thereby minimizing the risk of omitting important predictors. We then applied backward selection to reduce the model, supported by a hybrid strategy that incorporated both statistical criteria and biological plausibility. For instance, variables known to be associated with hantavirus or HEV from prior literature were retained even if not statistically significant in our dataset. This approach is also used in epidemiological research for identifying key factors associated with outcomes while maintaining interpretability (Bugeza et al., 2024; Rachmawati et al., 2024).  In addition, we performed goodness-of-fit tests (e.g., Hosmer-Lemeshow) in our final models to ensure that these models had acceptable performance and did not indicate signs of overfitting or poor specification.  To enhance clarity, we have revised the Methods – Data analysis and Discussion sections of the manuscript to explain this rationale and to acknowledge the limitations of stepwise approaches.  Line 281 – 284 (Methods): *“In the initial model, we included both statistically significant variables with p-values ≤ 0.05 and biologically plausible variables with p-values > 0.05 to minimize the risk of missing important factors. We then applied a backward stepwise regression approach to identify the best-fitting models.”*  Line 594 – 603 (Discussion)*: “Fourth, we used backward stepwise selection for multivariable modelling. While valuable for generating a parsimonious model in this exploratory study, this approach can increase risk for type 1 errors and exclusion of relevant but non-significant predictors [104]. To mitigate these concerns, we forced variables deemed biologically highly relevant, identified in the causal framework, to remain in the model. This strategy reflected the exploratory nature of our study and aligned with guidance that stepwise methods can be appropriate for early-stage investigations aimed at identifying potential predictors [105–107]. Future studies with larger samples and confirmatory designs should consider a full-model specification or Bayesian approaches to improve model stability and inference.”*  References:  Thayer JD (2002) Stepwise regression as an exploratory data analysis procedure. <https://files.eric.ed.gov/fulltext/ED464932.pdf>  Dohoo I, Martin W, Stryhn H. Veterinary Epidemiologic Research. 2nd ed. Charlottetown, PEI: VER Inc.; 2010. ISBN-13: 978-0-919013-60-5 ; Pages: 367  Chowdhury MZI, Turin TC. Variable selection strategies and its importance in clinical prediction modelling. Fam Med Community Health. 2020;8(1):e000262. Published 2020 Feb 16. doi:10.1136/fmch-2019-000262  Bugeza JK, Roesel K, Mugizi DR, Alinaitwe L, Kivali V, Kankya C, Moriyon I, Cook EAJ. Sero-prevalence and risk factors associated with occurrence of anti-Brucella antibodies among slaughterhouse workers in Uganda. PLoS Negl Trop Dis. 2024 Mar 18;18(3):e0012046. doi: 10.1371/journal.pntd.0012046. PMID: 38498555; PMCID: PMC10977895.  Rachmawati E, Riskiyah, Novindra QA, Syarifah NA, Aisy NR. Association between lifestyle factors and hypertension control in Indonesian primary healthcare settings: A cross-sectional study. Malays Fam Physician. 2024;19:18. Published 2024 Jun 12. doi:10.6084/m9.figshare.24878427.v1 |
| Abstract  Line 26: Report the type of blood sample. | We revised as suggested (line 26 – 27).  *“…207 provided serum samples for serological testing for hantavirus and HEV antibodies…”* |
| Line 36: Clarify the reference category for “wildlife farming activities only.” | We clarified as suggested (line 35 – 37)  *“Hantavirus seropositivity was significantly associated with engaging only in wildlife farming, and not participating in other activities such as hunting, trading, slaughtering, processing, guano collection, or consumption.”* |
| Introduction  The last paragraph mentions the “distinct wildlife trade dynamics” of Lao Cai and Dong Nai. It would be helpful to have some description of these dynamics here in the Introduction. How do the two regions differ and how might these differences affect exposure? | We thank the reviewer for this suggestion. We agree that understanding the differences between Lao Cai and Dong Nai is important for interpreting exposure pathways. To maintain focus and reduce redundancy, we have removed the phrase “distinct wildlife trade dynamics” from the Introduction section and instead expanded the description in the Study location subsection of the Methods. This revised text below explains how both provinces are involved in wildlife farming, while also highlighting regional differences in trade scale, species, and potential exposure routes.  Line 142 – 160: *“Lao Cai and Dong Nai represent two ecologically and economically distinct regions with different patterns of wildlife farming and trade. Lao Cai, a mountainous province bordering China, is characterized by small-scale wildlife farming integrated with hunting, home processing, and informal trade…Dong Nai, in contrast, is a lowland province in southern Vietnam with a high concentration of commercial wildlife farms. Farmers in both provinces may be exposed through contact with farmed wildlife, but in Lao Cai, additional risks may arise from hunting and butchering, while in Dong Nai, risks may extend along the commercial chain.”* |
| Methods What percent of farms in Dong Nai were recruited for the study? What were the characteristics of the farms selected for the study? | We have added the following clarification to the Study location section of the Methods.  Line 164 – 167: *“The selected farms varied in size and purpose, including small-scale household operations, medium-sized, and larger-scale commercial farms. While some farms focused solely on wildlife farming, others were also involved in trading, slaughtering, processing, harvesting bat guano, or consumption.”*  Line 178 – 179: *“In Dong Nai, we recruited 164 wildlife farms from six selected districts, accounting for 24.5% (164/ 669) of the province’s wildlife farms.”* |
| It is unclear how many participants were recruited from each farm. Were the samples relatively similar across farms? If not, how did they differ? | We appreciate the reviewer’s comment. To clarify, we recruited one participant per farm, resulting in a total of 210 participants from 210 distinct wildlife farms. This approach ensured independence across observations and avoided within-farm clustering. Each participant was either the farm owner or the individual most actively involved in daily wildlife farming activities.  We have clarified this point in the revised Methods section.  Line 182 – 190: *“All study participants were adult wildlife farm owners or workers aged 18 or older who provided informed consent. The participants were required to have at least six months of continuous involvement in wildlife farming to ensure sufficient exposure time for potential hantavirus or HEV infection and subsequent antibody development. Individuals not directly involved in the farming of wild animals, or with a known history of confirmed hantavirus or HEV infection, or receiving immunosuppressive therapy, were excluded. Eligible individuals who declined participation were replaced by other consenting participants. We recruited one participant per farm, typically selecting the owner or the individual most actively engaged in daily farming tasks.”* |
| Did the authors consider another level of nestedness in their model for farm? It is unclear how many participants were recruited from each farm, but a three-level model with both farm and province as random effects might be appropriate. | Thank you for the comment. As noted in the Methods section (line 280), we included province as a random effect to account for geographic clustering and variation in wildlife farming practices across regions. Since only one participant was recruited per farm, there was no within-farm clustering, and thus it was not necessary to include farm as an additional random effect. |
| Discussion:  Lines 450-452: Please include a reference for this statement (if possible): "In Vietnam, it is very common that local people consume uncooked wild meat and wildlife Products as they believe in the health benefits and exoticism of wild meat." | We added references as suggested  Line 508: Ref. 88 and 89  Shairp R, Veríssimo D, Fraser I, Challender D, MacMillan D. Understanding Urban Demand for Wild Meat in Vietnam: Implications for Conservation Actions. PLoS One. 2016 Jan 11;11(1):e0134787. doi: 10.1371/journal.pone.0134787. PMID: 26752642; PMCID: PMC4709058.  Huong VT, Hoa NT, Horby P, Bryant JE, Van Kinh N, Toan TK, Wertheim HF. Raw pig blood consumption and potential risk for Streptococcus suis infection, Vietnam. Emerg Infect Dis. 2014 Nov;20(11):1895-8. doi: 10.3201/eid2011.140915. PMID: 25340391; PMCID: PMC4214319. |
| **Reviewer #2** |  |
| General Comments: Investigation on risk factors for specific infections are of general importance for risk mitigation strategies to prevent disease in future. The two analyzed viruses – hantavirus and HEV – are important zoonotic antigens and wildlife farmers are generally considered to be of risk of these zoonotic infections. Although the results of the study are mainly of confirmational nature, the data are of general interest and might contribute to further develop specific prevention strategies. However, several aspects need to be addressed before publication of the manuscript.  First of all, the transmission pathways of HEV are insufficiently described, which might result in misinterpretation of findings. HEV can be grouped into four main human-pathogenic genotypes, of which only genotypes 3 and 4 are zoonotic and infect animals, especially pigs and wild boars. In contrast, genotypes 1 and 2 only infect humans and cannot be zoonotically transmitted. It is therefore misleading to include case numbers, which are derived from genotype 1 and 2 infection (see Ref. 26), directly before mentioning animal infections (lines 67-71), and mix all together. The situation is even more complicated as another different virus species, ratHEV, can infect rats and in some cases zoonotically transmitted to humans. Therefore:  - these genotypes/virus species, their transmission pathways and reservoirs has to be described in more detail  - the situation regarding the presence of these genotypes and ratHEV in Vietnam should be mentioned  - the specificity of the applied serologic test for detecting antibodies of the different HEV genotypes/virus species has to be included  - it would also be important to discuss the results in light of the genotypes/virus species host specificity, e.g. by comparing farmers keeping wild boar or bamboo rats vs. those keeping civets or bats. | We sincerely thank the reviewer for the constructive comments. We have revised the manuscript to address all four points raised:   - Point 1 and 2: Genotypes, virus species, transmission pathways, and reservoirs, as well as the presence of HEV genotypes and rat HEV in Vietnam: We have added the information to the Introduction section as suggested.   Line 74 – 86: “*HEV is a major public health concern, with an estimated 20 million infections occurring globally each year, accounting for over three million symptomatic cases and approximately 56,600 deaths worldwide [32,33]. HEV is divided into four main genotypes that cause human diseases (genotypes 1, 2, 3, and 4) with distinct host ranges and transmission pathways [32–34]. Genotypes 1 and 2 infect only humans via faecal-oral route, while genotypes 3 and 4 are transmitted through contact with infected animals or consumption of contaminated food or water [31–34]. Previous studies have isolated genotypes 3 and 4 in humans and various species of wild animals, including wild boars, rodents, and deer, which are all commonly farmed, indicating zoonotic transmission [20,35–40]. Additionally, rat HEV, a novel HEV strain that causes human hepatitis, has been detected in rats [41], and serological evidence of its infection has been observed in humans in Vietnam [42]. Suspected routes of rat HEV include contact with animal excreta, contaminated food or water, and environmental exposure [43].”*   - Point 3: Specificity of the serological test: We have added the information to the Method and Discussion section for clarification:   Line 243 – 245 (Method)*: “The Wantai HEV IgG assay targets the open reading frame 2 (ORF2) capsid protein, allowing detection of prior HEV exposure across all genotypes [63].”*  Line 586 – 594 (Discussion): *“Moreover, the Wantai HEV IgG ELISA, while capable of detecting antibodies against the highly conserved ORF2 capsid protein of all HEV assays, does not distinguish among its strains, which include zoonotic (genotypes 3 and 4), rat HEV, and other genotypes of HEV… Further research employing genotype-specific or molecular assays can provide more information on typing the virus, thereby mitigating the risk of misinterpreting zoonotic prevalence and associated risk factors.”*   - Point 4: Discussion of results in light of host specificity: We thank the reviewer for this thoughtful comment. We added our findings to the results section and added some discussion about this point:   Line 382 – 383 (Results): *“Our analysis did not find a significant association between the species farmed and seropositivity for either hantavirus or HEV”.*  Line 559 – 565 (Discussion): *“Our finding of no significant association between farmed wildlife species and hantavirus or HEV seropositivity may reflect the shared exposures across farms in our study, such as environmental contamination or similar farming practices. Previous studies reported higher seroprevalence in individuals handling specific animals, such as rats for hantavirus [103] or wild boars for HEV [80]. Future studies with larger sample sizes for different species, incorporating molecular detection from animals and environmental samples, could help clarify the role of specific host species in viral transmission dynamics.”* |
| The discussion is very long, also repeating already mentioned facts (e.g., in 1st paragraph) and should be condensed to the necessary information. | We thank the reviewer for this helpful comment. We have carefully revised the Discussion section to eliminate redundancy, particularly:   - In the first paragraph about AP and TP, we removed the explanation of the difference between AP and TP, which was already described in the Methods section (line 427 – 432) - In the second and fourth paragraphs about hantavirus and HEV prevalence, instead of comparing with many countries, we kept some relevant comparisons (line 433 – 440 and line 452 – 464) - In the tenth paragraph about dual seropositivity explanation, we removed the explanation of virus transmission pathways that were already mentioned in the introduction (line 566 – 572) - Moved the risk factors and consequences of viruses from the Discussion to the Introduction section (line 101 – 103 and line 87 – 95, respectively) |
| L. 26: It would be helpful to insert the animal species kept by the farmers (bats, bamboo rats, civets, wild boars) into the abstract | We added as suggested:  Line 26: *“A cross-sectional study was conducted among 210 wildlife farmers in Lao Cai and Dong Nai provinces in Vietnam who raised bats, bamboo rats, civets, and wild boars.”* |
| - L. 40-41: An explanation on the finding with the protective clothing should be inserted as the conclusions (improved biosecurity, proper hygiene, …) are confusing as they stand now. Please insert “…although their proper use was not assessed” in l. 41, or similar. | We revised as suggested.  Line 41 - 42: added *“…although their proper use was not assessed”* |
| - L. 65-71: see General Comments on HEV genotypes and species, their animal reservoirs, transmission pathways and occurrence in Vietnam. | Revised as suggested  See our responses for general comments (Point 1 and 2) above |
| - L. 187-191: see General Comments on specificity of the test for HEV genotypes and species. | Revised as suggested  See our responses for general comments (Point 3) above |
| - L. 282: read … (IgG and/or IgM) … | Revised as suggested  Line 342: *“…hantavirus (IgG and/or IgM) and…”* |
| - L. 382-383: Has there been an increased number of illnesses in the Dong Nai province, which might indicate a hantavirus outbreak? | To our knowledge, no hantavirus outbreaks have been officially reported in Dong Nai province during the study period or in recent years. We have added a statement in the Discussion line 451 to clarify that:  *“…and no hantavirus outbreaks have been officially reported in Dong Nai during the study period.”* |
| - L. 399-406: see General Comments on HEV genotypes/species and animal reservoirs. | We thank the reviewer for this helpful suggestion. In response, we have explicitly mentioned the zoonotic nature of HEV genotypes 3 and 4, as well as the potential health consequences of hantavirus and HEV infections, to better frame the rationale and significance of our study. This information has been added to the Introduction section.  Line 87 – 95: *“Both hantavirus and HEV infections may cause severe consequences, with hantavirus mortality rates ranging from <1% to 15% [44], and HEV mortality rates are notably high among pregnant women infected with genotypes 1 or 2, while HEV genotype 3 and 4 infections can range in severity from asymptomatic to fulminant hepatitis [45,46]. Similarly, rat HEV infections in humans can range from mild, self-resolving illnesses to life-threatening acute liver failure and chronic infections in individuals with weakened immune systems [47].”* |
| - L. 407-408: it should be considered that antibodies can persist for more than one year. Therefore, they might originate from illness a longer time ago. | We revised as suggested.  Line 466 – 468: *“It should be noted that antibodies, particularly IgG, can persist for years after infection. Therefore, they may have originated from an illness that occurred previously.”* |
| - L. 413-418: Did the hantavirus IgM-positive persons reported hantavirus-specific symptoms? | We thank the reviewer for this important question. In response, we have added a summary of self-reported symptoms among seropositive participants to the Results section. These include symptoms reported by individuals who tested positive for hantavirus IgG, hantavirus IgM, and HEV IgG.  Line 366 – 375: *“Among the 18 hantavirus IgG-positive participants, 8 (44.4%) reported experiencing symptoms, most commonly fatigue (n = 5), fever (n = 3), cough (n = 3), and headache (n = 2). Less frequently reported symptoms included shortness of breath, difficulty breathing, and sneezing/runny nose. Of the four hantavirus IgM-positive individuals, only one person reported symptoms consistent with acute hantavirus infection, including fever, fatigue, and cough, while the others were asymptomatic. Among the 55 HEV IgG-positive participants, 21 (38.2%) reported symptoms, including headache (n = 14), fatigue (n = 12), cough (n = 10), fever (n = 7), and shortness of breath (n = 5). Less commonly, participants reported sneezing or runny nose (n = 3), difficulty breathing (n = 3), vomiting (n = 2), diarrhoea (n = 2), muscle pain (n = 2), as well as weakness, rash, and hoarseness.”* |
| - L. 428: Please specify if “affected by HEV” relates to HEV infection or HEV-induced disease. | We revised as suggested.  Line 486: *“…men were more likely to have been infected with HEV,…”* |
| - L. 487-504: Please include in discussion the finding of Ref. 72, which described evidence for the benefit of protective gloves during disemboweling of wild boars, for preventing HEV infection. | We revised as suggested.  Line 553 – 556: *“Nevertheless, Schielke et al. [90] provided evidence that wearing protective gloves during the disembowelling of wild boars significantly reduced the risk of HEV infection among hunters, highlighting that PPE can be effective when used correctly in high-risk activities.”* |
| - l. 513: see General Comments on HEV genotypes and species and their transmission pathways. | We revised as suggested.  See our response for general comments.  We sincerely thank the reviewer for the thoughtful comments. We moved and revised the explanation of HEV genotype and its transmission pathways to the Introduction section to provide appropriate background and strengthen the motivation for our research. (line 74 – 86) |
